# Regular laboratory testing and patient survival among patients undergoing maintenance hemodialysis: a Korean nationwide cohort study

**DOI:** 10.1038/s41598-023-45502-8

**Published:** 2023-10-26

**Authors:** Do Hyoung Kim, AJin Cho, Hayne Cho Park, Bo Yeon Kim, Miri Lee, Gui Ok Kim, Jinseog Kim, Young-Ki Lee

**Affiliations:** 1 Department of Internal Medicine, Kangnam Sacred Heart Hospital, Hallym University College of Medicine, Singil-ro, Yeongdeungpo-gu, Seoul, 07441 Korea; 2 Hallym University Kidney Research Institute, Seoul, Korea; 3 Healthcare Review and Assessment Committee, Health Insurance Review and Assessment Service, Wonju, Korea; 4 Division of Quality Assessment 1, Health Insurance Review and Assessment Service, Wonju, Korea; 5 Division of Quality Assessment Management, Health Insurance Review and Assessment Service, Wonju, Korea; 6 Department of Bigdata and Applied Statistics, Dongguk University, Gyeongju, Korea

**Keywords:** Haemodialysis, End-stage renal disease

## Abstract

Routine laboratory tests are regularly performed in patients undergoing maintenance hemodialysis (HD) to detect anemia, chronic kidney disease-mineral bone disorders, and cardiovascular disease. More frequent laboratory tests may be associated with better outcomes. However, there is little evidence supporting a specific monitoring interval. This study evaluated the impact of regular laboratory testing on mortality in Korean patients undergoing maintenance HD. We used HD quality assessments, and National Health Insurance Service claims data from October to December 2015. In HD quality assessment, 22 tests are recommended every 1–6 months. A total of 34,950 patients were divided into two groups based on the regularity of laboratory testing. A Cox proportional hazards model was used to assess the effects of regular laboratory tests on patient mortality during a mean follow-up duration of 53.7 months. The proportion of patients with and without regular laboratory testing was 85.6% (n = 29,914) and 14.4% (n = 5036), respectively. Patients who underwent regular laboratory testing had a longer dialysis duration, lower serum phosphorus levels and diastolic blood pressure, and higher hemoglobin and single-pool Kt/V levels than those who did not. After adjusting for demographic and clinical parameters, regular laboratory testing independently reduced mortality risk (hazard ratio, 0.90; 95% confidence interval 0.85–0.95; *P* < 0.001). Regular laboratory testing was associated with a decreased mortality risk among patients undergoing HD. Management of end-stage kidney disease-related complications based on laboratory tests can improve survival.

## Introduction

Chronic kidney disease (CKD) is irreversible kidney damage associated with progressive loss of kidney function over time. The loss of kidney function results in a wide range of complications. Patients with end-stage kidney disease (ESKD) experience numerous complications, including anemia, fluid and electrolyte disorders, CKD-mineral bone disorder (CKD-MBD), and cardiovascular diseases. Routine laboratory testing is critical for managing complications in patients with ESKD on dialysis.

Routine laboratory tests increase the likelihood of detecting treatable problems; however, more frequent tests may increase healthcare utilization and costs^1,2^. There is no evidence to guide optimal laboratory testing intervals. The Kidney Disease: Improving Global Outcomes (KDIGO) guidelines for anemia in CKD provides an ungraded guideline to sample hemoglobin (Hb) monthly and ferritin and transferrin saturation (TSAT) every 3 months in patients undergoing hemodialysis (HD)^3^. In 2017, the KDIGO included an ungraded recommendation to test for serum calcium and phosphate levels every 1–3 months and parathyroid hormone (PTH) levels every 3–6 months in patients with CKD stage 5D^4,5^. The KDIGO guidelines also recommend that hepatitis C virus (HCV) testing be performed every 6 months in in-center dialysis patients^6^. The Korean Society of Nephrology (KSN) has also developed clinical practice guidelines for optimal HD according to international standards^7^. In the KSN clinical practice guidelines, test recommendations are based on expert opinions, given that most HD facilities perform blood tests monthly.

Few studies have demonstrated how the frequency of routine laboratory tests for patients undergoing HD affects patient outcomes. A Canadian study showed that monthly routine blood tests were not associated with reduced mortality risk compared with testing every 6 weeks^2^. This study aimed to evaluate the effect of regular laboratory testing on mortality in patients undergoing maintenance HD.

## Results

### Baseline characteristics of participants based on regular laboratory testing

**Figure 1:**
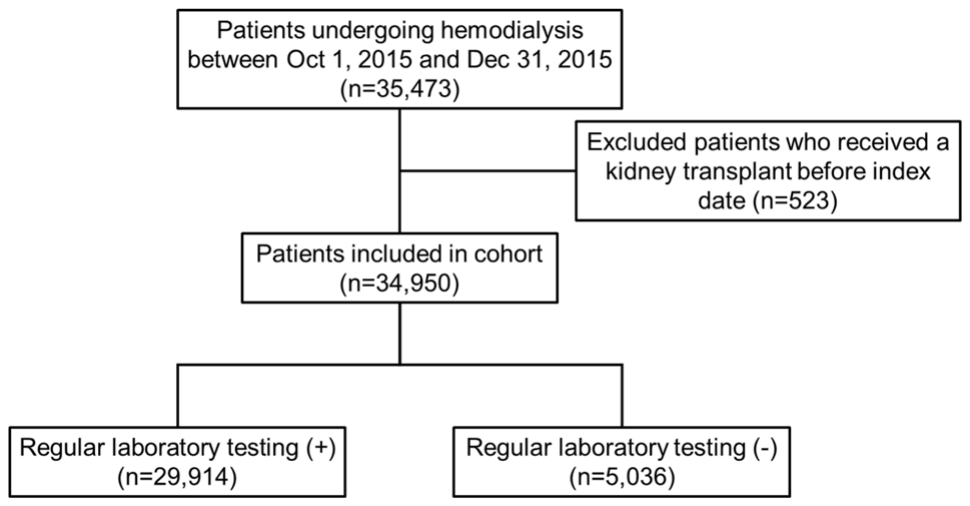
Flowchart of the study. Flowchart demonstrates the selection process of study population.

This study included 34,950 patients treated at 798 facilities. The proportion of patients who underwent regular laboratory testing and those who did not were 85.6% (n = 29,914) and 14.6% (n = 5036), respectively (Fig. 1). The baseline characteristics of the patients, based on regular laboratory tests, are shown in Table 1. Longer dialysis duration and lower rates of hypertension were observed in patients who underwent regular laboratory testing than in those who did not. Patients who underwent regular laboratory testing also had lower serum phosphorus levels and diastolic blood pressure. Plasma Hb levels and single-pool Kt/V were significantly higher in patients who underwent regular laboratory testing than in those who did not.

**Table 1:** Baseline characteristics of the subjects based on regular laboratory testing.

Variable	Total (n = 34,950)	Regular laboratory testing ( +) (n = 29,914)	Regular laboratory testing (−) (n = 5036)	SMD
HD facilities	798	649	149	
Mean age, y	60.15 ± 12.82	60.18 ± 12.84	59.97 ± 12.71	0.049
Male,	20,543 (58.8)	17,482 (58.4)	3061 (60.8)	0.048
Dialysis vintage, y	5.61 ± 5.10	5.77 ± 5.07	4.64 ± 5.14	0.223
Diabetes mellitus	21,491 (61.5)	18,240 (61.0)	3251 (64.6)	0.074
Hypertension	29,665 (84.9)	25,192 (84.2)	4473 (88.8)	0.135
IHD	12,091 (34.6)	10,248 (34.3)	1843 (36.6)	0.049
Heart failure	5125 (14.7)	4386 (14.7)	739 (14.7)	< 0.001
CVA	3117 (8.9)	2643 (8.8)	474 (9.4)	0.020
Atrial fibrillation	1851 (5.3)	1612 (5.4)	239 (4.7)	0.029
Hemoglobin	10.72 ± 0.85	10.73 ± 0.84	10.63 ± 0.90	0.119
Albumin, g/dL	3.99 ± 0.35	3.99 ± 0.34	3.99 ± 0.37	0.020
Calcium, mg/dL	8.99 ± 0.81	8.99 ± 0.81	9.00 ± 0.86	0.011
Phosphorus, mg/dL	4.94 ± 1.33	4.91 ± 1.32	5.11 ± 1.36	0.147
Single pool Kt/V	1.55 ± 0.28	1.56 ± 0.27	1.50 ± 0.29	0.226
Medicaid	8031 (23.0%)	6872 (23.0%)	1159 (23.0%)	0.001
BMI, kg/m^2^	22.37 ± 3.39	22.38 ± 3.40	22.33 ± 3.33	0.015
SBP, mmHg	141.19 ± 15.55	140.96 ± 15.46	142.51 ± 15.98	0.098
DBP, mmHg	77.55 ± 9.56	77.28 ± 9.60	79.16 ± 9.15	0.200
PP, mmHg	63.63 ± 14.12	63.68 ± 14.17	63.35 ± 13.79	0.024

Data expressed as number (percent), mean ± standard deviation,

BMI, body mass index; CVA, cerebrovascular accident; DBP, diastolic blood pressure; IHD, ischemic heart disease; HD, hemodialysis; Kt/V, hemodialysis adequacy; PP, pulse pressure; SBP, systolic blood pressure; SMD, standardized means difference.

### Crude rate of all-cause mortality according to routine laboratory tests

**Figure 2:**
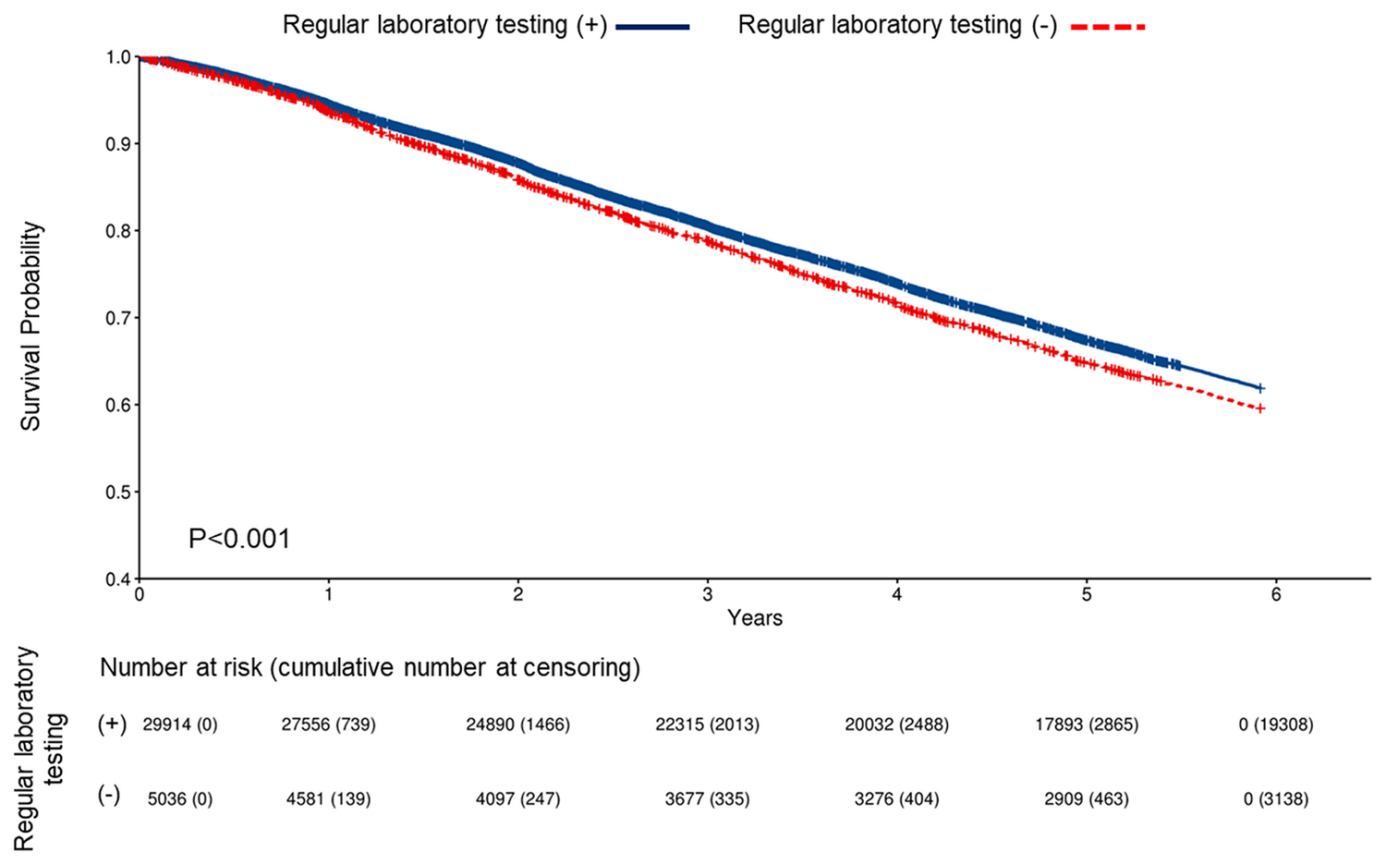
Kaplan–Meier survival curves according to regular laboratory testing. A total of 12,504 deaths occurred during 53.7 ± 23.0 months. After censoring 3515 (10.1%) cases who received kidney transplantation, patients with routine laboratory tests showed better survival compared to patients without routine laboratory tests (*P* < 0.001).

A total of 12,504 deaths (35.8%) occurred within 53.7 ± 23.0 months. The crude death rate was 79.9 patients per 1,000 person-years. The crude mortality rate ratio was lower in patients with regular laboratory tests than in patients without regular laboratory tests (79.0 vs. 85.8 patients per 1000 person-years, *P* < 0.001) (Table 2). 3515 (10.1%) patients were censored after kidney transplantation. The Kaplan–Meier survival curve showed a lower mortality risk among patients with regular laboratory tests than in those without (Fig. 2).

**Table 2:** Comparison of crude all-cause mortality rate (/1000 patient-years) according to regular laboratory testing.

	Total (n = 34,950)	Regular laboratory testing (+) (n = 29,914)	Regular laboratory testing (−) (n = 5036)	*P*-value
Number of death during follow-up	12,504	10,606	1898	0.002
Total follow up person-years	156,418	134,288	22,131	0.001
Crude death rate (/1000 person-years)	79.9	79.0	85.8	< 0.001

### Regular laboratory testing independently decrease mortality risk

The Cox proportional hazards model was used to identify the risk factors associated with patient mortality. In the univariate analysis, old age, male sex, lower BMI, presence of comorbidities (diabetes mellitus, hypertension, ischemic heart disease, heart failure, cerebrovascular accident, and atrial fibrillation), lower plasma Hb, serum albumin, calcium, and phosphorus levels, and lower single-pool Kt/V were associated with a higher mortality risk (Supplementary Table 2). In addition, regular laboratory testing was associated with lower mortality (hazard ratio (HR), 0.92; 95% confidence interval (CI), 0.88–0.97; *P* < 0.001) (Table 3). When we adjusted for age, sex, dialysis vintage, and BMI (Model 1), regular laboratory testing was still an independent predictor for patient mortality (HR, 0.89; 95% CI 0.85–0.94; *P* < 0.001). When we adjusted for comorbid conditions in addition to factors included in Model 1 (Model 2), regular laboratory testing remained an independent risk factor for patient mortality (HR, 0.89; 95% CI 0.85–0.94; *P* < 0.001). Finally, we conducted a multivariate analysis by additionally adjusting for plasma Hb, serum albumin, phosphorus, calcium levels, and spKt/V in Model 2 (Model 3), which revealed that regular laboratory testing was an independent risk factor for all-cause mortality (HR, 0.90; 95% CI 0.85–0.95; *P* < 0.001).

**Table 3:** Multivariable Cox regression analysis for the patient mortality according to regular laboratory testing.

	HR	95% CI	*P* value
Unadjusted	0.92	0.88–0.97	< 0.001
Model 1*	0.89	0.85–0.94	< 0.001
Model 2†	0.89	0.85–0.94	< 0.001
Model 3‡	0.90	0.85–0.95	< 0.001

CI, confidence interval; HR, hazard ratio.

*Model 1: adjusted age, sex, dialysis vintage, and body mass index.

^†^Model 2: adjusted Model 1 + history of diabetes mellitus, hypertension, ischemic heart disease, heart failure, cerebrovascular accident, and atrial fibrillation.

^‡^Model 3: adjusted Model 2 + plasma hemoglobin, serum albumin, calcium, phosphorus, and single pool Kt/V.

### Subgroup analysis for all-cause mortality according to regular laboratory testing

**Figure 3:**
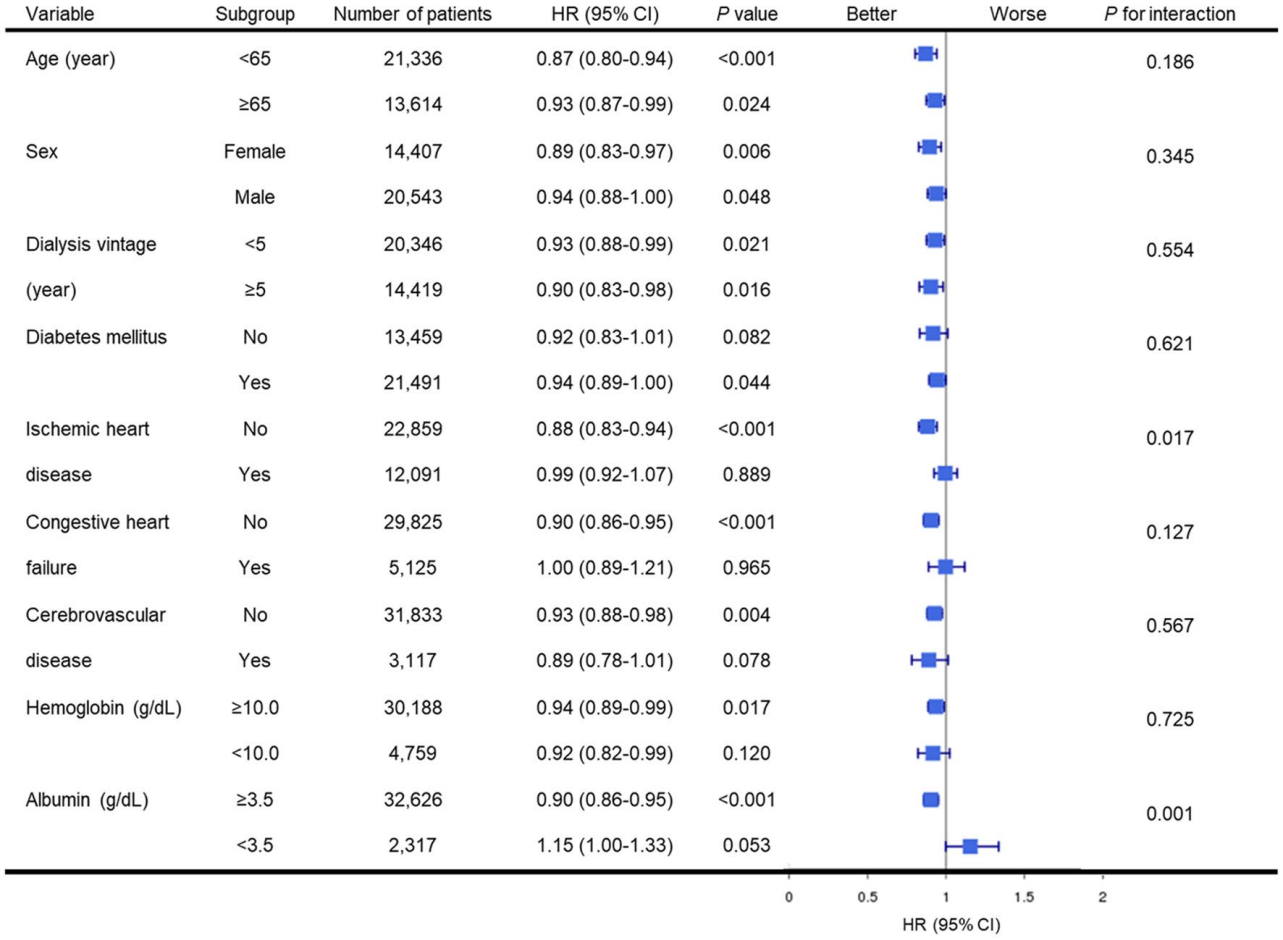
Forest plot depicting the relative risk of patient mortality according to regular laboratory testing in different subgroups. Regular laboratory testing was associated with decrease in mortality in patients regardless of their age, gender or presence of dialysis vintage. However, regular laboratory testing was associated with decreased mortality in patients without ischemic heart disease, congestive heart failure or cerebrovascular disease; with diabetes mellitus; or in patients without anemia or hypoalbuminemia. CI, confidence interval; HR, hazard ratio.

We divided the patients into subgroups to compare the mortality risk using regular laboratory testing according to subgroups. Regular laboratory testing was associated with decreased mortality in patients, regardless of age, gender or dialysis vintage (Fig. 3). Additionally, regular laboratory testing was associated with decreased mortality in patients without ischemic heart disease, congestive heart failure, or cerebrovascular disease; with diabetes mellitus; or in patients without anemia or hypoalbuminemia.

## Discussion

This study showed that in-center HD patients with routine laboratory testing had improved survival rates compared to patients without routine blood testing. Moreover, higher plasma Hb levels and lower diastolic blood pressure were observed in patients who underwent regular laboratory testing than in those who did not. Dialysis adequacy was significantly higher in patients who underwent regular laboratory testing.

Which routine tests and what frequencies are appropriate for patients undergoing HD? There is no solid evidence to determine the optimal frequency of surveillance tests for patients undergoing maintenance HD. Several studies have attempted to determine the optimal interval between laboratory tests to maintain parameters within guideline targets. Gaweda et al. investigated the optimal frequency of Hb sampling and concluded that weekly measurements resulted in better anemia management^9^. Greenberg et al. compared quarterly PTH monitoring with monthly monitoring and found that monthly measurements achieved better KDOQI target of PTH levels^10^. However, Yokoyama et al. revealed that weekly monitoring of calcium and monthly monitoring of PTH levels were helpful if serum marker levels were above the target range and that there was no evidence that frequent measurement was helpful if mineral levels were already within the target range^11^. Most recently, Chidiac et al. revealed that the median number of interventions per year based on routine laboratory tests did not exceed six times for all parameters and suggested testing for Hb, serum calcium, and phosphate every 2 months and for PTH twice a year^12^.

In Korea, the HD Quality Assessment recommends that 22 laboratory tests (hemoglobin, platelet count, total protein, albumin, glucose, urea, creatinine, uric acid, sodium, potassium, total calcium and phosphorus every month, iron status, PTH, HbA1C (for people with diabetes), and chest radiograph every 3 months, viral markers, and electrocardiogram every 6 months) be performed regularly. Although there is no evidence to support the items and intervals for these laboratory tests, the frequency of routine laboratory tests is based on clinical practice guidelines^3–6,13,14^ or empirical evidence. Potassium control in patients with CKD is important because it is associated with an increased risk of arrhythmias and sudden cardiac death^15^. The frequency of potassium testing in patients undergoing hemodialysis is unclear. Chidiac suggested monitoring serum potassium levels every 2 months^12^. However, Bianchi et al. have argued that serum potassium levels should be measured monthly^15^. The KDIGO recommends that it is reasonable to measure HbA1C twice a year for long-term glycemic control monitoring in people with diabetes and up to four times a year when the glycemic goal is not reached, or antihyperglycemic therapy is changed^13^. The KDIGO also recommends evaluation for heart disease, including electrocardiography, at the start of dialysis and annually after that^14^. Also, recommended monitoring intervals for serum calcium and phosphate were every 1–3 months, and for PTH were every 3–6 months in KDIGO^5^. In the evaluation of anemia, KDIGO recommends that Hb be evaluated at least every 3 months in patients with hemodialysis without anemia, and every month in patients with anemia^16^. In KDIGO, the intervals for Hb, calcium, phosphorus, PTH, and HbA1c tests are broadly defined as, for example, 1–3 months or 3–6 months, but in the HD quality assessment, the intervals are set at minimum intervals. The KSN clinical practice guidelines are similar to the HD quality assessment recommendations. These guidelines recommend complete blood count (CBC), liver function tests (total protein and albumin levels), routine chemistry tests (blood urea nitrogen, creatinine, sodium, potassium, calcium, phosphate, uric acid, and glucose) monthly, iron status, PTH, HbA1C, and chest radiographs quarterly, hepatitis virus markers, and electrocardiography semiannually^7^. Most HD facilities in Korea conduct blood tests monthly because these tests are uncomplicated and inexpensive. In this study, 85.5% of patients underwent regular laboratory tests, and this percentage is gradually increasing^17^.

It is uncertain whether more frequent laboratory blood tests confer better outcomes in patients undergoing HD. No randomized controlled trials or prospective observational studies have reported test items’ and interval outcomes in patients undergoing HD. Thomas et al. examined whether monthly blood testing was associated with better clinical outcomes than testing every 6 weeks in Ontario, Canada^11^. While 70% of the programs conducted monthly blood tests as a common practice, the remainder adopted a 6-week interval^11^. They found that monthly sampling of blood parameters, compared with sampling at 6-week intervals, was not associated with lower mortality, fewer cardiovascular events, or fewer healthcare visits in patients undergoing maintenance HD. We demonstrated that regular laboratory testing was associated with a lower risk of death in patients undergoing maintenance HD. Regular laboratory tests can detect patients’ problems early. Treatment of ESKD-related complications and modification of dialysis prescriptions based on laboratory test results could lead to better survival and quality of life in patients undergoing HD^7^. However, it is necessary to consider the costs associated with testing, medical devices, and care providers^7^.

This study had several strengths. The HIRA is a national system, and this study included a large sample pool. In addition, the follow-up period was longer than those reported in previous studies. The results were rigorously examined using an HD quality assessment program to categorize the laboratory test frequency accurately. Previous studies examining blood testing intervals focused on surrogate parameters, such as the percentage of patients reaching guideline-based biochemical or hematologic targets. In contrast, this study examined long-term patient mortality.

This study had several limitations. First, therapeutic interventions based on routine laboratory testing and the percentage of patients reaching guideline-based targets were not analyzed. Second, the occurrence of morbidities, such as cardiovascular disease, infection, malnutrition, and vascular access problems, could not be examined. Third, the causes of death and disease-specific mortality were not analyzed. Fourth, the analysis was performed without adjusting the facility-level characteristics of hospitals involved in the HD quality assessment. In addition, the observation period for regular laboratory testing was short to generalize our results. Further analysis with long-term observation should be warranted. Finally, the cost-effectiveness of laboratory monitoring at short intervals was not considered; further research is required to determine whether regular laboratory testing is cost-effective.

In conclusion, routine laboratory testing was associated with a lower mortality risk in patients undergoing maintenance HD. Managing ESKD-related complications based on laboratory tests can lead to better survival. Randomized controlled trials are needed to define the frequency of laboratory tests that optimally benefits the health of patients undergoing HD and has the greatest impact on the use of healthcare resources.

## Methods

### Study population and data collection

This longitudinal observational cohort study included Korean patients undergoing maintenance HD. HD quality assessment data from the Health Insurance Review and Assessment Service (HIRA) from 2015 were used, and mortality data were collected between January 2016 and November 2021. Patients aged ≥ 18 years who received HD treatment more than twice weekly were enrolled. Patients enrolled in this study were outpatient at each HD facility. HD quality assessment data were not collected for patients hospitalized or not followed-up during the assessment period. Patients who had received a kidney transplant before index date were excluded from the analysis (Fig. 1).

HD quality assessments were based on the data entered into each HD facility and collected using a web-based data collection system^8^. The collected data included 12 measures in three domains (structure, process, and outcome) (Supplementary Table 1). Demographic and clinical data were obtained from the HIRA database. Demographic factors collected included age, sex, dialysis duration, body mass index (BMI), and health insurance status. The medical comorbidities of the participants were identified using the International Classification of Disease (ICD-10) codes from the health insurance claims database from January to December 2015. The categories of comorbidities were diabetes mellitus (E10–14), hypertension (I10–13 and I15), ischemic heart disease (I20–25), congestive heart failure (I50), and cerebrovascular disease (I60–64 and I69). Systolic and diastolic blood pressure were measured before dialysis. Laboratory parameters, including plasma Hb, serum albumin, calcium, and phosphorus levels, were collected monthly during the study period. The average values of three measurements were used in the analysis. Dialysis adequacy was evaluated using single-pool Kt/V. The date of death was defined as the date of insurance loss.

This study was conducted in accordance with the Declaration of Helsinki and was approved by the Institutional Review Board (IRB) of Hallym University Kangnam Sacred Heart Hospital (No. HKS 2021-11-043). The Hallym University Kangnam Sacred Heart Hospital Institution Review Board waived the need for written informed consent from the patients because the study participants were not identified.

### Regular laboratory testing and outcomes

HD quality assessment required 22 laboratory tests performed at regular intervals (Table 4). Laboratory parameters should be tested monthly, including Hb, platelets, total protein, albumin, glucose, urea, creatinine, uric acid, sodium, potassium, total calcium, and phosphorus levels. HD quality assessment also requires testing for ferritin, transferrin saturation (TSAT [iron/total iron-binding capacity]), PTH, hemoglobin A1c (HbA1c) (for people with diabetes), and chest radiography every 3 months. Tests for hepatitis B surface antigen (HBsAg), hepatitis B surface antibody (anti-HBs), hepatitis C virus antibody (anti-HCV), and electrocardiography are recommended every 6 months. HD quality assessment accepts only the tests performed within the specified interval for each laboratory test. The testing interval of each laboratory test was evaluated on monthly bases regardless of the specified date. For example, if hemoglobin was tested on March 7th, next test should be conducted between April 1st and April 30th. Therefore, HD quality assessment is based on intervals of months. The evaluation period for each laboratory test differed according to the test interval (Table 4). The cases which adhere to regular laboratory testing were defined as follows;

1. Testing done every month for 3-month evaluation period for 1-month interval laboratory tests.
2. Testing done at least twice with 3-month interval within 6-month evaluation period for 3-month interval laboratory tests.
3. Testing done at least twice with 6-month interval within 1-year evaluation period for 6-month interval laboratory tests.

**Table 4:** The test items and intervals of HD quality assessment.

Test items	Intervals	Evaluation period
Hemoglobin, platelet count, total protein, albumin, glucose, urea, creatinine, uric acid, sodium, potassium, total calcium, phosphorus	At least monthly	Oct 2015–Dec 2015
Iron, TIBC, ferritin, PTH, HbA1c (in patients with DM), chest radiograph	At least every 3 months	July 2015–Dec 2015
HBs Ag, anti-HBs, anti-HCV, electrocardiography	At least every 6 months	January 2015–Dec 2015

DM, diabetes mellitus; HbA1c, hemoglobin A1c; PTH, parathyroid hormone; TIBC, total iron binding capacity.

If the tests were conducted at intervals other than above, they were considered non-adherence cases to regular laboratory test. Among these laboratory tests, test results for serum Hb, albumin, total calcium, phosphorus, and single-pool Kt/V were obtained. Data on frequency were collected for other laboratory tests. The primary outcome measure was all-cause mortality. Patients who received kidney transplants during the follow-up period were censored at the time of kidney transplantation.

### Statistical analyses

The study population was divided into two groups based on the regularity of laboratory tests. Chi-square tests for categorical variables and independent t-tests for continuous variables were used to compare baseline characteristics. Continuous variables were described as means and standard deviations, and categorical variables were expressed as frequencies and percentages. Kaplan–Meier analysis and log-rank tests were used to compare the risk of mortality between the groups. A multivariate-adjusted Cox proportional hazards model was used to determine whether regular laboratory testing was an independent predictor of mortality. Model 1 was adjusted for age, male sex, dialysis vintage, and BMI. Model 2 was adjusted for medical comorbidities (diabetes mellitus, hypertension, ischemic heart disease, heart failure, cerebrovascular accident, and atrial fibrillation) and the factors included in Model 1. Model 3 was adjusted for all demographic and clinical parameters (plasma Hb, serum albumin, calcium, phosphorus, and single-pool Kt/V). Finally, subgroup analyses were performed to define the relative mortality risk according to regular laboratory testing. Subgroup analysis was performed according to age group (< 65 vs. ≥ 65 years), sex, dialysis vintage (< 5 vs. ≥ 5 years), comorbidities, serum Hb (< 10.0 vs. ≥ 10.0 g/dL), and albumin levels (< 3.5 vs. ≥ 3.5 g/dL). All statistical analyses were performed using R version 4.0.2 (R Foundation for Statistical Computing, http://www.r-project.org/). The standardized mean difference was calculated, and P < 0.05 was considered statistically significant.

### Supplementary Information

Supplementary Tables.

## Data Availability

The data that support the findings of this study are available from HIRA, but restrictions apply to the availability of these data, so they are not publicly available. Data are, however, available from the authors upon request and with permission from HIRA. Permission to access the data can be requested through Gui Ok, Kim (turtle52@hira.or.kr), HIRA’s Division of Quality Assessment Management, co-author of this study.
