# Supplementary material for: Regular laboratory testing and patient survival among patients undergoing maintenance hemodialysis: a Korean nationwide cohort study
Source: Sci Rep. 2023 Oct 26;13:18360. doi: 10.1038/s41598-023-45502-8 (PMC10603026; doi:10.1038/s41598-023-45502-8)
Supplement: Supplementary file 1 — Supplementary Tables. [file 41598_2023_45502_MOESM1_ESM.docx]

**Regular laboratory testing and patient survival among patients undergoing maintenance hemodialysis: A Korean nationwide cohort study**

Do Hyoung Kim ^1, 2^, AJin Cho ^1, 2^, Hayne Cho Park ^1, 2^, Bo Yeon Kim ^3^, Miri Lee ^4^, Gui Ok Kim ^5^, Jinseog Kim^6^, Young-Ki Lee ^1, 2*^

^1^ Department of Internal Medicine, Kangnam Sacred Heart Hospital, Hallym University College of Medicine, Seoul, Korea

^2^ Hallym University Kidney Research Institute, Seoul, Korea

^3^ Healthcare Review and Assessment Committee, Health Insurance Review and Assessment Service, Wonju, Korea

^4^ Division of Quality Assessment 1, Health Insurance Review and Assessment Service, Wonju, Korea

^5^ Division of Quality Assessment Management, Health Insurance Review and Assessment Service, Wonju, Korea

^6^ Department of Bigdata and Applied Statistics, Dongguk University, Gyeongju, Korea

**Short Title: Regular laboratory testing and patient survival**

***Corresponding Author:**

Young-Ki Lee, MD, PhD

Department of Internal Medicine

Hallym University College of Medicine, Kangnam Sacred Heart Hospital

Singil-ro, Yeongdeungpo-gu

Seoul 07441, Korea

Tel: +82-2-829-5214

E-mail: km2071@hallym.or.kr

**Supplement Tables** **1. Components of hemodialysis quality assessment**

| Domains | | HD quality assessment  (12 measures) | Equation |
| --- | --- | --- | --- |
| Structural | Personnel | (1) Percent of doctors specialized in HD | [Σ (employed days of each doctor specialized in HD)/Σ (employed days of each doctor)] × 100 |
|  |  | (2) Percent of nurses with ≥ 2 years experience in HD | [Σ (employed days of each nurse with ≥ 2 years experience in HD)/Σ (employed days of each nurse)] × 100 |
|  |  | (3) Number of HD performed per doctor per day | Total number of HD/Σ (working days of each doctor) |
|  |  | (4) Number of HD performed per nurse per day | Total number of HD/Σ (working days of each nurse) |
|  | Equipment | (5) Satisfaction of the minimum required number of isolated HD equipment for hepatitis B patients | Minimum required number of isolated HD equipment  = number of hepatitis B patients/[(3*days of nocturnal HD) + (2*days of day-time HD)]/3 |
|  |  | (6) Availability of emergency equipment in HD room | Emergency equipment: Oxygen supply, suction, Endotracheal intubation kit, electrocardiogram, defibrillator |
|  | Facilities | (7) Satisfaction of the minimum required frequency of water quality tests | Minimum required frequency of tests  * Bacterological assay: monthly, 1/12 of total HD equipments  * Endotoxin assay: every 3 months  * Chemical assay: annually |
| Procedural | HD adequacy | (8) Satisfaction rate of the minimum required frequency of HD adequacy test | [Number of patients satisfied with minimum required frequency of HD adequacy test/Total number of ambulatory HD patients] × 100  * Minimum frequency: every 3 months |
|  | Vascular access | (9) Satisfaction rate of the minimum requirement for vascular access stenosis monitoring | [Number of patients satisfied with minimum required frequency of vascular access stenosis monitoring/ Total number of ambulatory HD patients] × 100  ※ Monitoring methods  * Monthly: static intra-access pressure ratio, ultrasound dilution technique, duplex ultrasonography, angiography  * Weekly: physical exam of vascular access |
|  | Regular tests | (10) Satisfaction rate of the required frequency of regular laboratory tests | [Number of patients satisfied with minimum required frequency of regular laboratory tests/ Total number of ambulatory HD patients] × 100 |
| Monitoring | spKt/V | (11) Satisfaction rate of HD adequacy | [Number of patients satisfied with HD adequacy/Total number of HD patients tested for HD adequacy] × 100  * HD adequacy: spKt/V ≥ 1.2 or URR ≥ 65% |
|  | Mineral bone disorder | (12) Satisfaction rate of calcium × phosphorus | [(The number of patients with calcium × phosphorus < 55)/Total number of HD patients tested for calcium and phosphorus during assessment period] × 100 |

HD, hemodialysis; spKt/V, single-pool Kt/V; URR, urea reduction ratio

**Supplement Tables 2. Mortality hazard ratio according to regular laboratory testing**

| **Variables** | **Univariate** | | **Multivariate** | | | | | |
| --- | --- | --- | --- | --- | --- | --- | --- | --- |
|  |  |  | **Model 1** | | **Model 2** | | **Model 3** | |
|  | **HR (95% CI)** | **P-value** | **HR (95% CI)** | **P-value** | **HR (95% CI)** | **P-value** | **HR (95% CI)** | **P-value** |
| **Regular laboratoty testing** | 0.92 (0.88-0.97) | <0.001 | 0.89 (0.85-0.94) | <0.001 | 0.89 (0.85-0.94) | <0.001 | 0.90 (0.85-0.95) | <0.001 |
| **Age, *y*** | 1.07 (1.06-1.07 | <0.001 | 1.07 (1.07-1.07) | <0.001 | 1.06 (1.06-1.07) | <0.001 | 1.06 (1.06-1.06) | <0.001 |
| **Male (vs. female)** | 1.17 (1.13-1.22) | <0.001 | 1.32 (1.27-1.36) | <0.001 | 1.27 (1.22-1.32) | <0.001 | 1.22 (1.16-1.27) | <0.001 |
| **Dialysis vinatge, *y*** | 0.99 (0.99-1.00) | <0.001 | 1.01 (1.01-1.02) | <0.001 | 1.02 (1.02-1.03) | <0.001 | 1.02 (1.02-1.03) | <0.001 |
| **BMI, *kg/m^2^*** | 0.97 (0.97-0.98) | <0.001 | 0.98 (0.97-0.98) | <0.001 | 0.97 (0.96-0.97) | <0.001 | 0.97 (0.96-0.97) | <0.001 |
| **Diabetes mellitus** | 1.91 (1.84-1.99) | <0.001 |  |  | 1.61 (1.55-1.68) | <0.001 | 1.58 (1.51-1.66) | <0.001 |
| **Hypertension** | 1.31 (1.24-1.38) | <0.001 |  |  | 1.00 (0.95-1.06) | 0.957 | 0.98 (0.93-1.04) | 0.591 |
| **IHD** | 1.64 (1.58-1.70) | <0.001 |  |  | 1.21 (1.17-1.26) | <0.001 | 1.19 (1.15-1.24) | <0.001 |
| **Heart failure** | 1.49 (1.42-1.56) | <0.001 |  |  | 1.15 (1.10-1.21) | <0.001 | 1.14 (1.09-1.20) | <0.001 |
| **CVA** | 1.90 (1.81-2.00) | <0.001 |  |  | 1.36 (1.29-1.44) | <0.001 | 1.32 (1.25-1.40) | <0.001 |
| **Atrial fibrillation** | 1.85 (1.73-1.97) | <0.001 |  |  | 1.29 (1.21-1.38) | <0.001 | 1.27 (1.18-1.36) | <0.001 |
| **Plasma Hb, *g/dL*** | 0.88 (0.86-0.89) | <0.001 |  |  |  |  | 0.91 (0.89-0.94) | <0.001 |
| **Serum albumin, *g/dL*** | 0.36 (0.34-0.37 | <0.001 |  |  |  |  | 0.57 (0.54-0.61) | <0.001 |
| **Serum calcium, *mg/dL*** | 0.92 (0.90-0.94) | <0.001 |  |  |  |  | 1.09 (0.06-1.12 | <0.001 |
| **Serum phosphorus, *mg/dL*** | 0.84 (0.83-0.85) | <0.001 |  |  |  |  | 1.03 (1.01-1.06) | 0.002 |
| **Single pool Kt/V** | 0.86 (0.80-0.93) | <0.001 |  |  |  |  | 0.73 (0.67-0.80) | <0.001 |

Abbreviations: BMI, body mass index; CI, confidence interval; CVA, cerebrovascular accident; IHD, ischemic heart disease; HD, hemodialysis; HR, hazard ratio; Kt/V, hemodialysis adequacy.
